# Regulating the Performance of Lithium-Ion Battery Focus on the Electrode-Electrolyte Interface

**DOI:** 10.3389/fchem.2020.00821

**Published:** 2020-09-04

**Authors:** Dongni Zhao, Shiyou Li

**Affiliations:** ^1^College of Petrochemical Technology, Lanzhou University of Technology, Lanzhou, China; ^2^Gansu Engineering Laboratory of Electrolyte Material for Lithium-Ion Battery, Lanzhou, China

**Keywords:** interfacial film, CEI film, advanced characterization, lithium-ion battery, mechanism

## Abstract

The development of lithium-ion battery (LIB) has gone through nearly 40 year of research. The solid electrolyte interface film in LIBs is one of most vital research topics, its behavior affects the cycle life and safety of LIBs significantly. Progress in understanding the interfacial layer on the negative and positive electrodes in LIBs has been the focus of considerable research in the past few decades, but there remains a number of problem to be understood at the fundamental level, and there is still a great deal of controversy regarding the composition and formation mechanism of the interfacial film. In this article, we summarize recent research conducted on the interfacial film in LIBs, including the film formation mechanism, the composition, and stability of the interfacial film on the positive electrodes (in both diluted and high-concentration electrolytes). And the methodologies and advanced techniques implemented for the characterization of the interfacial film. Finally, we put forward some of the future development direction for the interfacial film and urgent problems that need to be solved.

## Introduction

In recent years, the demands on lithium-ion batteries has increased, due to their applicability in hybrid and electric vehicles. The requirements for such application manifest in the a cycle life and safety features needed for the successful application of the device. The operational mechanism for the lithium-ion battery works through the movement of electric charge through an external circuit to balance the shuttle movement of lithium-ions in the main structures of the cathode and anode of the device (Mizushima et al., 1980; Yazami and Touzain, 1983; Goodenough and Kim, 2010; Goodenough, 2018; Han et al., 2019). The positive and negative electrodes are kept apart by a separator to avoid short circuiting, and are surrounded with an aprotic non-aqueous electrolyte. When the anodic operating voltage of the cell is close to 0 V, elemental lithium is stored; when the cathodic voltage of the cell exceeds 3 V, lithium-ion is stored (Hightower et al., 2000; Lu et al., 2011; Suo et al., 2012; Zheng et al., 2013). The reaction of the aprotic electrolyte on the electrode is thermodynamically unstable. Therefore, understanding the active electrochemical and chemical reactions on the electrode-electrolyte interface is the key to the development of a stable, high-efficiency lithium-ion battery. These reactions occuring on the electrode will affect the composition, the microstructure, and the properties of the interfacial film, while the nature of the interfacial film will determine the coulombic efficiency, cycle life, and operational safety during the operational life of the battery.

Cyclic carbonate-based electrolytes are widely used in lithium-ion batteries, such as ethylene carbonate (EC), and they go through reduction or oxidation reactions on the surface of negative or positive electrodes, to form the well-known electrode-electrolyte interface film (EEI). The EEI is permeable to lithium-ions in the electrolyte but not conductive to the electrons on the electrode, and its formation can impede the continuation of the reduction and oxidation of the electrolyte, and the irreversible consumption of lithium-ions during cycling. However, the formation of a non-uniform EEI film may result in uneven lithium deposition and cause the formation of lithium dendrites, which in turn leads to internal short circuiting and failure in the battery. The interface film formed on the positive electrode is incomprehensible in composition and properties compared to that forming on the negative electrode (Verma et al., 2010; Xing et al., 2011; Leung, 2013; Gallego et al., 2014). The study of the cathode electrode interface (called as CEI film) film is the key to reducing the activity between the electrolyte and positive electrode material, which will affect the life and safety of the battery, because the exothermic reaction between the positive electrode material and the flammable electrolyte generates a large amount of heat and cause thermal runaway.

**Figure 1:**
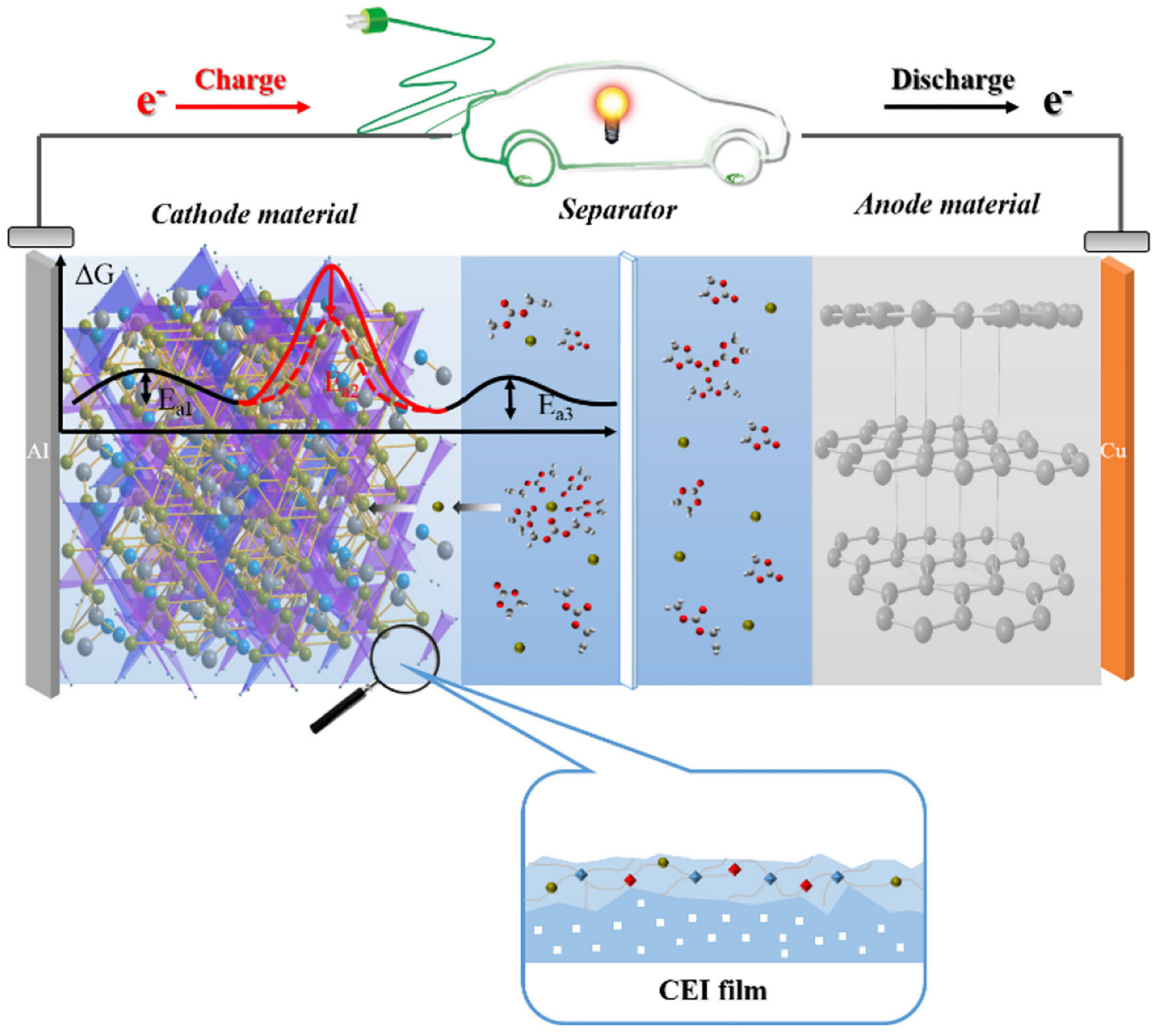
Schematic diagram of the research structure of the lithium-ion battery interface film.

Although many advanced researches have been made in studying the properties of interfacial films in LIBs, and many advanced equipment and technologies are continuously being used to investigate and characterize their nature and composition, many aspects of the properties of interfacial films remain in need of further exploration. For example, the film formation mechanism and the role of composition in the behavior of the interfacial film and performance of the battery have not been thoroughly studied. Figure 1 gives a schematic diagram of the research structure of the positive electrode interface film for lithium-ion batteries.

This article summarizes the progress of research related to CEI films in LIBs positive electrode in the past decade. Firstly, the thermodynamic factors of the redox reaction on the positive and negative electrodes are reviewed and discussed to understand the basic principles behind them. Then, the formation mechanism, composition, and stability of the interfacial film on the positive electrode are mainly discussed. Finally, the main characterization methods of the interfacial film are summarized, and the opportunities and techniques that can be used to obtain further insight on the formation mechanism and the composition of the interfacial film, as well as the correlation between the properties of the interfacial film and battery performance, are also put forward and discussed.

## Effect of Thermodynamic Factors on the Formation of Interfacial Films

**Figure 2:**
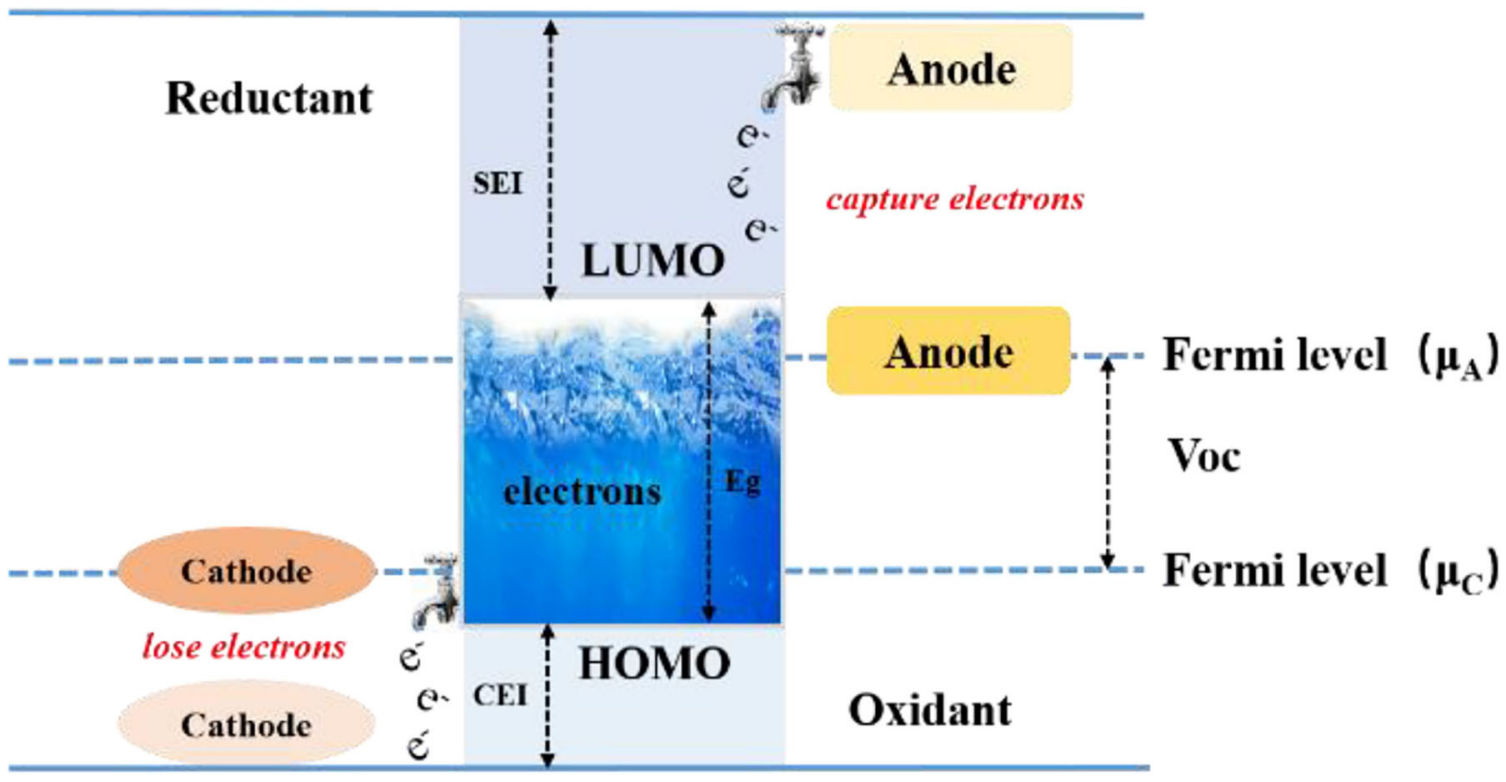
Schematic drawing formation mechanism of SEI (Goodenough and Kim, 2010).

Goodenough et al. described the relationship between the Fermi level of the positive and negative electrodes in a lithium-ion battery as well as the solvent and electrolyte HOMO (highest occupied molecular orbital) and LUMO (lowest unoccupied molecular orbital) in the electrolyte (shown in Figure 2) (Borodin et al., 2013; Goodenough, 2018). The difference between the Fermi energies of the electrodes and the HOMO and LUMO energy levels of the electrolyte controls the thermodynamics and the driving forces behind the formation of the electrode surface interfacial film (Goodenough and Kim, 2010). When the LUMO level of the organic solvent or electrolyte lithium salt is lower than the Fermi level of the negative electrode, electrons will be injected into the LUMO orbit by that driving force, causing the electrolyte component to be reduced; when the HOMO level is higher than Fermi energy level of the positive electrode, electrons are driven into the positive electrode, causing the solvent or lithium salt to be oxidized. The solvent or lithium salt is reduced or oxidized at the surface of the electrode during charging, and a portion of the resulting substance that is insoluble in the electrolyte will be deposited on the surface of the negative electrode or the positive electrode (Goodenough and Kim, 2010). These substances can conduct lithium-ions transferred from the electrolyte due to the presence of lithium-ions, but at the same time they are insulating to electrons from the electrode, which prevents the electrode from further reacting with the electrolyte. We will give below a detailed review of the dynamic driving force behind the formation of the interfacial film, in relation to the instability of the electrolyte on the electrode surface.

### Storage Electrode Potential of Lithium

**Figure 3:**
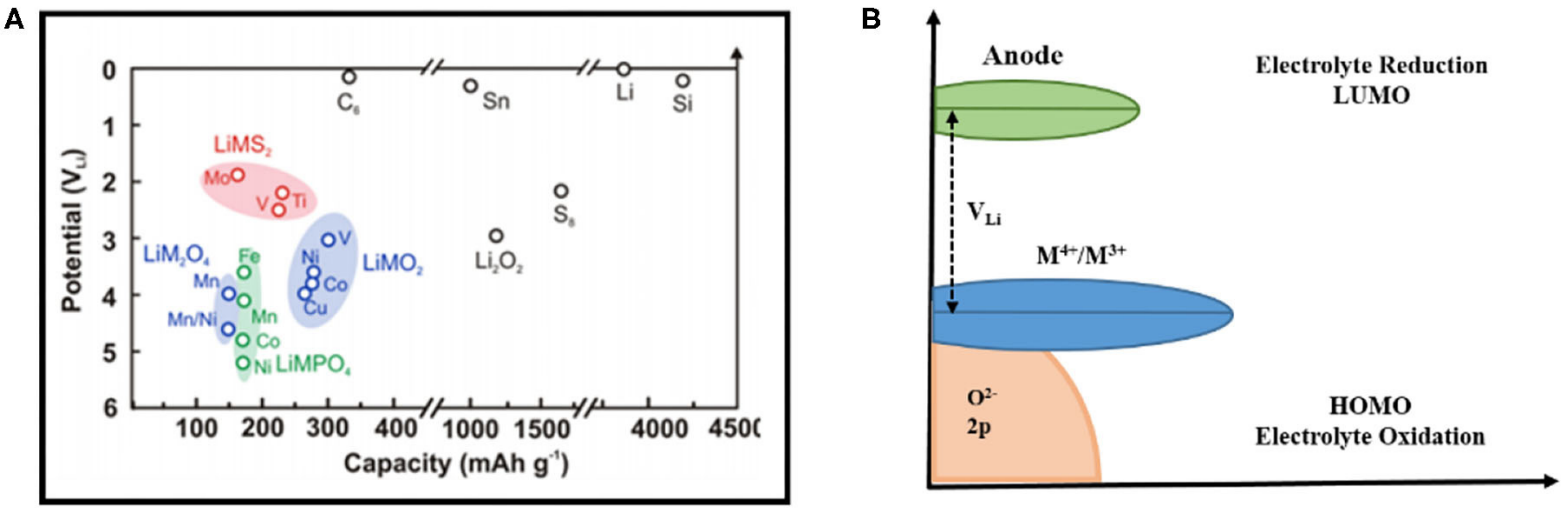
**(A)** Comparison of potential and theoretical capacity of several lithium-ion battery lithium storage cathode materials (Zhang et al., 2001); **(B)** The difference between the HOMO/LUMO orbital energy level of the electrolyte and the Fermi level of the electrode material controls the thermodynamics and driving force of interface film growth (Goodenough and Kim, 2010).

The electrode potential of lithium metal corresponds to the average electron energy level at the top of its valence band (electron transfer energy level or redox electron energy of materials). The difference in electrochemical potential between the positive and negative electrodes gives the thermodynamic battery voltage change, the kinetic effects come from the battery assembly, current rates, electrode configuration, and electrolyte not from their standard redox potential. The most common negatively charged sheets of metallic lithium and graphite store lithium at 0 and ~0.1 V, respectively, and their Fermi level is above the estimated LUMO level of the electrolyte (Figure 3A; Zhang et al., 2001). Therefore, there is a driving force on the surfaces of these negative electrodes to promote the electrochemical reduction of the electrolyte. The surface of the electrode is passivated by the stable, electronically insulating, lithium-ion-conducting surface film formed on the interface, thereby preventing the electrode surface from coming into direct contact with the aprotic electrolyte.

The potential of lithium transition metal compounds such as oxides, sulfides, and phosphates (Figures 3A,B) is lower than the reduction potential of the aprotic electrolyte, and their electrochemical potentials are largely determined by the redox energy of the transition metal ion (Yazami and Touzain, 1983; Xu et al., 1999; Egashira et al., 2001). On the other hand, by replacing the M-O skeleton in LiMO_2_ (M refers to metal element) with the inductive effect of M-O-P, the redox energy of the transition metal ion may be further reduced (i.e., the lithium intercalation potential is further increased, Figure 3A) (Zhou et al., 2004; Wolfenstine and Allen, 2005; Leung, 2013). There is closer energy level between oxidation potential of high voltage energy storage materials and HOMO level of electrolyte. Examples include materials such as Li_1−x_Mn_1.5_Ni_0.5_O_4_ (Ni^4+^/Ni^2+^ at 4.7 V), Li_1−x_NiPO_4_ (Ni^3+^/^2+^ at 5.2 V) and even Li_1−x_CoO_2_ (x <0.7, voltage above 4.5 V), which are shown in Figure 3A, and have the thermodynamic driving force for the oxidation of the electrolyte on the electrode surface (Wolfenstine and Allen, 2005; Ménétrier et al., 2008). Recent research has focused on some new lithium storage material, including high-capacity layered composites or solid solutions such as yLi_2−x_MnO_3_**∙**(1–y) Li_1−x_MO_2_ (M = Ni, Co, Mn) (Thackeray et al., 2007; Croy et al., 2013), lithium-rich material such as Li_1+y_M_1−y_O_2_ (Koga et al., 2012; Ates et al., 2015) and Li_2_MO_3_ (M = Ru, Mn, Ti, Sn) (Ménétrier et al., 2008; Sathiya et al., 2015). In addition to the use of transition metal ion redox to store lithium, the redox reactions of oxyanions are also used compared to LiCoO_2_ has a real capacity of 140 mAh g^−1^, with more than 230 mAh g^−1^ of reversible capacity, where the electron energy in the d orbital of the transition metal drops below that in the p orbital of the oxygen anion (Thackeray et al., 2007; Koga et al., 2012; Croy et al., 2013). The redox reaction of these oxide anions may cause the production of higher active species such as the peroxide ion $O_{2}^{2-}$-, molecular oxygen, and superoxide compounds (${\text{O}}_{2}^{-}$ is produced by the reduction of O_2_ at voltages below ~3 V), that will chemically react with an aprotic electrolyte to form a surface film (Xu et al., 2011).

### Estimation of the Electrolyte LUMO/HOMO Energy Levels

**Figure 4:**
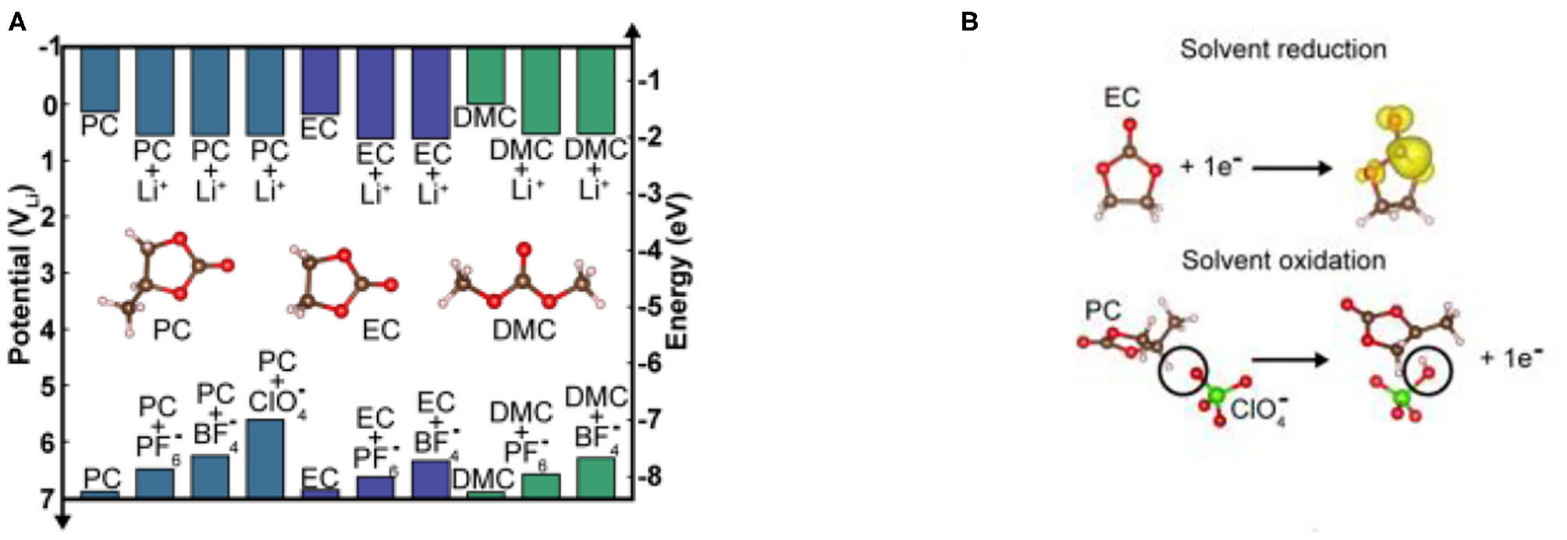
**(A)** Calculated reduction and oxidation energy levels of common solvents and solvated salts for Li-ion battery. **(B)** Reaction formulae used for calculating redox levels under ideal conditions (Gauthier et al., 2015) (reproduced from ref. 5 with permission from Springer Nature, copyright 2016).

The first adiabatic electron transfer and the nature of electrolyte itself can be used to estimate the HOMO/LUMO levels of electrolyte. The solvent molecules and Li^+^ solvation or anion solvation complexes are used to describe the oxidation and reduction processes of pure solvents and solvated lithium salts, respectively (Xing et al., 2011; Leung, 2013). The reduction potential (relative to the LUMO energy levels) is obtained by density functional theory (DFT) calculations with adding a continuous pole model (Vollmer et al., 2004; Leung, 2013). For example, the carbonate solvents EC, PC, and DMC, which are commonly used in lithium-ion batteries, are found to have LUMO energy close to the Li^+^/Li pair (shown in Figure 4A; Vollmer et al., 2004; Leung, 2013). Through calculation, it was found that several homologs of pure carbonate solvents such as PC, EC, and DMC, are not particularly large in their LUMO values, which indicates that the number of carbon chains has a small effect on the LUMO value of carbonate groups. The main reason is related to the electron transfer occurring mainly in the carbonate group (shown in Figure 4B). When lithium-ions are included in the calculation, the calculated LUMO energy level will be reduced by ~0.5 eV relative to the pure solvent, and the stability will therefore decrease, and the reduction reaction will occur on the lithium or graphite anode (Figure 4A). In addition to the influence of lithium-ions on the solvent LUMO level, the role of lithium salt anions such as ${\text{PF}}_{6}^{-}$, ${\text{ClO}}_{4}^{-}$, and ${\text{BF}}_{4}^{-}$ should also be considered, which requires further computational studies.

The calculated HOMO energy level of the pure-carbonate solvent is lower than the Fermi level of the cathode material, and its oxidation potential is close to 7 V (Zhang et al., 2001). When the anion of the salt is added to the solvent, the calculated HOMO level of the electrolyte is increased by ~1.5 eV (the potential is about 5.5 V), which reduces the thermal stability of the electrolyte relative to the electrochemical oxidation reaction, and then proton transfer between anions causes the oxidation of the electrolyte (Xing et al., 2011). The thermal stability of the electrolyte decreases in the order of ${\text{PF}}_{6}^{-}>$${\text{BF}}_{4}^{-}>$${\text{ClO}}_{4}^{-}$, and the complexation of ${\text{PF}}_{6}^{-}$ with the solvent is the most resistant to oxidation (Figure 4A).

Although DFT calculations predict that the electrolyte solvent is not easily oxidized when the potential is lower than ~5.5 V, carbonate-based electrolytes can be electrochemically oxidized in the range of 4.5–6.5 V according to reported experimental results (Xu et al., 1999; Egashira et al., 2001). Tests on the catalytic activation of different electrodes show that these contradictions may be caused by the presence of impurities leading to the change in the redox potential during the experiment, and to the inaccuracies associated with DFT calculations. The data reported in Figure 4A shows the clear trend of using the calculated HOMO/LUMO energy levels of the solvent molecules to judge the electrochemical stability window of electrolyte, and ignoring the interaction between the solvent and the electrode surface.

In addition to the electrochemical reduction and oxidation of the electrolyte described in Figures 4A,B, the electrolyte may decompose through other chemical reactions, and may compete with these electrochemical reactions simultaneously, which will affect the composition of the interfacial film formed on the electrode (Endo et al., 1998). We will introduce next in this article the influence of the electrochemical and chemical reactions of different positive electrodes on the composition and structure of the interfacial film, and how the electrode composition and decomposition affect them.

## Mechanism of Film Formation on the Cathode Surface (Including Dilute and Concentrated Solution)

### Mechanism of Positive Film Formation in Dilute Solution Electrolyte

There is no thermodynamic driving force for the electrochemical oxidation of the electrolyte on most traditional positive electrode materials (Figure 4A). There are currently three generally accepted mechanisms that describe the formation of the CEI film on the positive electrode (Li et al., 2008; Kempaiah et al., 2019; Gründer and Lucas, 2020):

(1) The drift mechanism, which considers the solid-electrolyte interface (SEI) film on the surface of the positive electrode to form through the reduction of the organic electrolyte on the negative electrode that is saturated with the electrolyte, and then diffused and deposited on the surface of the positive electrode material (Aurbach, 2000; Ostrovskii et al., 2001);

(2) The nucleophilic reaction mechanism, according to which electrophilic solvent molecules (such as EC or DMC) in the organic electrolyte are formed by a nucleophilic reaction with the negatively charged positive electrode materials or the interfacial film forms when the solvent reacts with the electrode, then the insoluble products from that reaction are deposited on the surface of the electrode (Wang et al., 2004a);

(3) The spontaneous reaction mechanism that relates the interfacial layer to the products from the reaction between electrolyte and positive electrode (Wang et al., 2004b; Wang and Chen, 2005).

Battery cycling under high voltage and high temperature will exacerbate the formation process of the interfacial film. Research on the positive electrode interfacial film is still in the development stage, and plenty of studies found that the film covering the surface of the positive electrode material is also incomplete, which caused by the continuous decomposition of the CEI film. There are three reasons to investigate the film formation on the positive electrode (Dupre et al., 2009): (1) the driving force of film formation. Compared with the negative electrode, the driving force for the positive electrode film formation is not easy to study. This is because of the structure of the negative electrode. Taking graphite as an example, the fragile structure of graphite is supported by weak Van der Waals forces, which allows its structure to easily co-embed and dissolve in the solvent, often leading to the failure of the battery. The coulombic forces and covalent bonds constituting the positive electrode material result in robust structure that is not particularly sensitive to the co-embedding of solvents; (2) the complex chemical composition on the surface of the cathode material makes it difficult to identify the electrochemical oxidation reaction of the electrolyte. The surface of most cathode materials originally has a film of Li_2_CO_3_ that is formed on the transition metal oxide, then subsequently reacts with the acid electrolyte to introduce new substances (Wang et al., 2003; Würsig et al., 2005; He et al., 2008; Dupre et al., 2009). Although quite a few of the products of these spontaneous reactions are interfacially mixed with the products of subsequent reactions, the irreversible electrochemical reaction and the phase change on the surface of the cathode will occur during the initial charging cycle, making the final interface different in chemical composition and morphology; (3) more importantly, the operation voltage of most cathode materials does not tend to be excessively higher than the limit of the oxidation potential of the electrolytic solution. Unlike with the negative electrode, the decomposition of the electrolytic solution at the positive electrode is not inevitable. However, with “5 V” positive electrode materials such as LiNi_0.5_Mn_1.5_O_4_ (4.6 V vs. Li^+^/Li) or LiCoPO_4_ (4.8 V vs. Li^+^/Li), the thermodynamic stability of the surface potential of the positive electrode becomes more positive compared to that of the components of the organic electrolyte, which Fermi level of the material is higher than the HOMO level of the electrolyte.

In general, the film on the surface of the positive electrode undergoes at least three processes: (1) a natural surface film forms during the electrode manufacturing process; (2) the natural surface film goes through a spontaneous chemical reaction when exposed to the electrolyte; (3) the products of steps (1) and (2) go through electrochemical rearrangement during the initial charging process.

Thomas et al. was the first to show the presence of an interfacial film on the surface of the cathode material, and their work was followed by a large number of studies that tried to characterize the structure of the film and speculate its origin (Thomas et al., 1985). The interfacial film on the positive electrode usually consists of chemical species surprisingly similar to the products of the decomposition of solvents and salts, similarly to the case of the products found on the negative electrode. The interfacial film on the positive electrode is mainly derived from the electrochemical chemically nucleophilic attack reaction on the electrode surface. Using *in situ* Fourier-transform infrared spectroscopy (FTIR), Wang et al. demonstrated the presence of carboxylate groups (O-C = O) on the surface of Li_1−x_CoO_2_ under cycling (Laudadio et al., 2019), while recently the oxidation of carbonate solvent on the surface of LiNi_x_MnyCo_1−x−y_O_2_ has also been developed by the *in situ* FTIR (Zhang et al., 2020). The use of lithium-rich layered compounds such as 0.5Li_2−x_MnO_3_ 0.5Li_1−x_Co_0.33_Ni_0.33_Mn_0.33_O_2_ or Li_1.2−x_Co_0.13_Ni_0.13_Mn_0.54_O_2_ as reported in numerous recent studies, will give rise to the further oxidation of the electrolyte by reaction with the O_2_ released during the charging process, and generate inorganic carbonates Li_2_CO_3_ (Yabuuchi et al., 2011). The use of *in-situ* monitoring techniques for probing the changes occurring on the surface of the cathode, like the use of *in-situ* synchronous XPS and XAS on Li_1−x_Mn_1.5_Ni_0.5_O_4_ neutron reflectometer and LiCoO_2_ or Li_1−x_Ni_0.2_Co_0.7_Mn_0.1_O_2_ (Browning et al., 2014; Yamamoto et al., 2014; Cherkashinin et al., 2015), offers the potential to provide new insight on the process of formation of the interfacial films on the surface of the positive electrode material, without any interference from the effect of the conductive agent and the binder. Also, the interface film of high-nickel ternary material LiNi_0.8_Co_0.1_Mn_0.1_O_2_ is also a recent research content (Hirbod and Xifei, 2019).

### Mechanism of CEI Film Formation in High-Concentration Electrolyte

**Figure 5:**
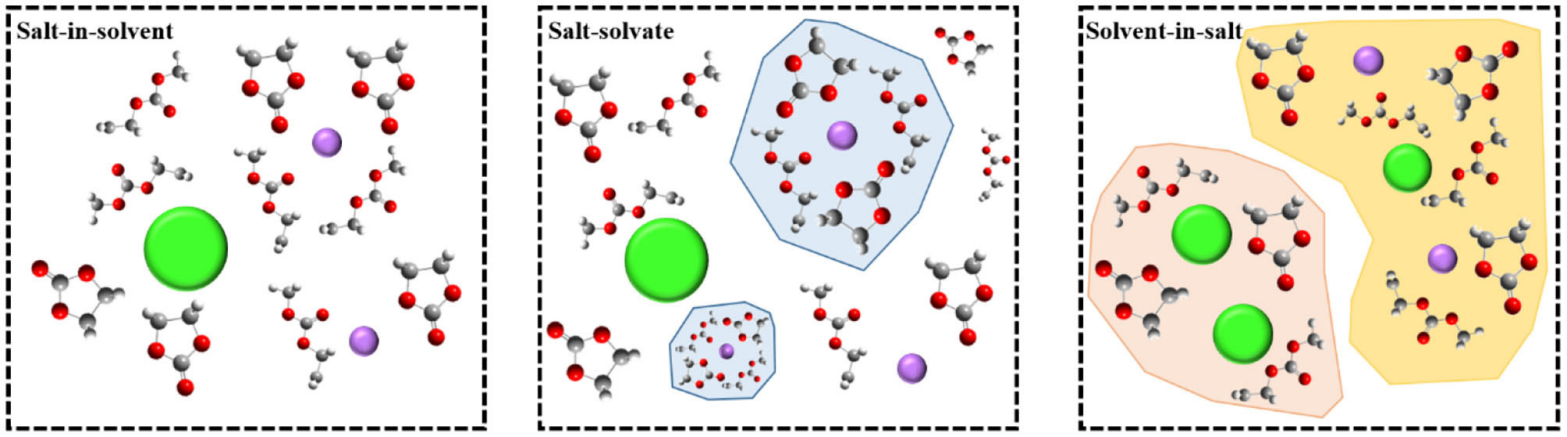
Schematic diagram of electrolytes classified by concentration (Borodin et al., 2020).

The mechanism of film-forming in high-concentration electrolytes is quite different from that of traditional diluted solution systems. The SEI film in high-concentration electrolytes is derived entirely from the anions, and the choice of anions will therefore directly determine the chemical properties of the interfacial film. Recently, and by defining the structure of ionic solvation, Borodin et al. temporarily divided all electrolytes into three different categories, instead of defining a limited concentration limit (Borodin et al., 2020). As shown in Figure 5, these categories are: (1) “salt-in-solvent” electrolyte, in which the number of solvent molecules is higher than that required for first-order solvation of all cations; (2) “salt-solvated” electrolyte, in which the number of solvent molecules is just enough to complete the main cations solvent layer, so most dissociated salts usually form stoichiometric solvates; (3) “solvent-in-salt” electrolyte, which is a super-concentrated electrolyte. These three categories provide a new idea for us to elucidate the formation mechanism of the interfacial film in high-concentration electrolytes. Although conventional electrolytes with molarity of 1.0 M belong to the “solvent-in-salt” category (Xu, 2014; Borodin et al., 2020), the latter two categories cover ultra-concentrated electrolytes, of which “salt solvation” is also commonly referred to as “solvated ionic liquid” to highlight its relationship with room temperature ionic liquids (RTIL), because of the lower proportion of “free” solvents. The main limitation on obtaining super-concentrated electrolytes is obviously the solubility of the salt in a given solvent, which is related to the melting point, disorder, and crystallization kinetics of the solvate, and can produce crystallinity and voids at salt concentrations. The special properties of the positive electrode surface brought about by the high concentration of an electrolyte are often apparent in the interfacial electrochemical behavior.

Among the “extraordinary” properties of concentrated electrolytes, there are reactions that may qualify as new interfacial chemical reactions different from those occurring in dilute solutions. This new interface has been recognized as the key to allow the electrochemical performance to reach extreme voltages. Examples on such electrolytes include ultra-concentrated ethers, sulfones, sulfites, nitriles, and water, which form a protective interfacial layer on the electrode material and allow the material to exhibit reversibility at high potentials (Xu, 2004; von Wald Cresce et al., 2012). All these new interfacial layers have the characteristics of the anions rather than those of the solvent molecules. Unlike dilute solutions, the interfacial chemical reaction is mainly the oxidative decomposition of solvent molecules. That new interfacial chemistry that relies on anions rather than solvent molecules, has lifted many of the traditional restrictions on electrolyte design, the most obvious of which being ethylene carbonate (EC), which is not an option in the manufacturing of any modern lithium-ion battery. The lack of solvent is mainly due to its key role in film formation on the surface of graphite negative electrodes (Xu, 2004; von Wald Cresce et al., 2012).

Although there is a correlation between the solvated shell of lithium-ion and the hypothetical transition state of the lithium ion-solvent co-embedded at the graphite interface, predicting the interfacial chemical reactions remains a challenge (von Wald Cresce et al., 2012). In general cases, it can be reasonably assumed that when an electrode potential reaches the threshold value of the decomposition potential of the electrolyte component, that is, when the interfacial chemical reaction begins, there may already be an interfacial structure existing in the so-called internal Helmholtz layer. This self-assembly of electrolyte components is enriched in some components and absent in others, and should be the intermediate parent entity that determines the final interface. Therefore, understanding the chemical composition and structural properties of an interface can open the door for predicting interfacial electrochemical reactions (Borodin et al., 2017; Suo et al., 2017; Vatamanu and Borodin, 2017; Yang et al., 2017, 2019; McEldrew et al., 2018). The same process occurs on the surface of the positive electrode. Molecular dynamics simulations show that the anion concentration in the inner layer of Helmholtz increases when the electrode is positively polarized (Vatamanu et al., 2012). When the electrolyte is based on a mixed solvent, such as the typical formulation of a commercial lithium-ion battery, and regardless of whether it is a negative electrode or a positive electrode, the preferential coordination of EC increases its chance of participating in the formation of SEI and CEI compared to DMC or other linear carbonates. When the salt concentration is ~1.0 M, both solvents and anions are observed in the internal Helmholtz layer near the surface of the positive electrode, but the super-concentrated electrolyte or ionic liquid will completely build up in the internal Helmholtz layer and disperse all the solvents molecules, causing them to cover the surface of the positive electrode, and preventing the possibility of their oxidation (McOwen et al., 2014; Borodin et al., 2017; Vatamanu and Borodin, 2017; McEldrew et al., 2018). The anionic structure plays a key role in determining whether or not adsorption occurs preferentially. For example, molecular dynamics simulations (Vatamanu and Borodin, 2017) found that TFSI has a greater advantage than trifluoromethanesulfonate (OTf) during positive electrode polarization, and this result was confirmed using infrared spectroscopy of the electrode surface.

**Figure 6:**
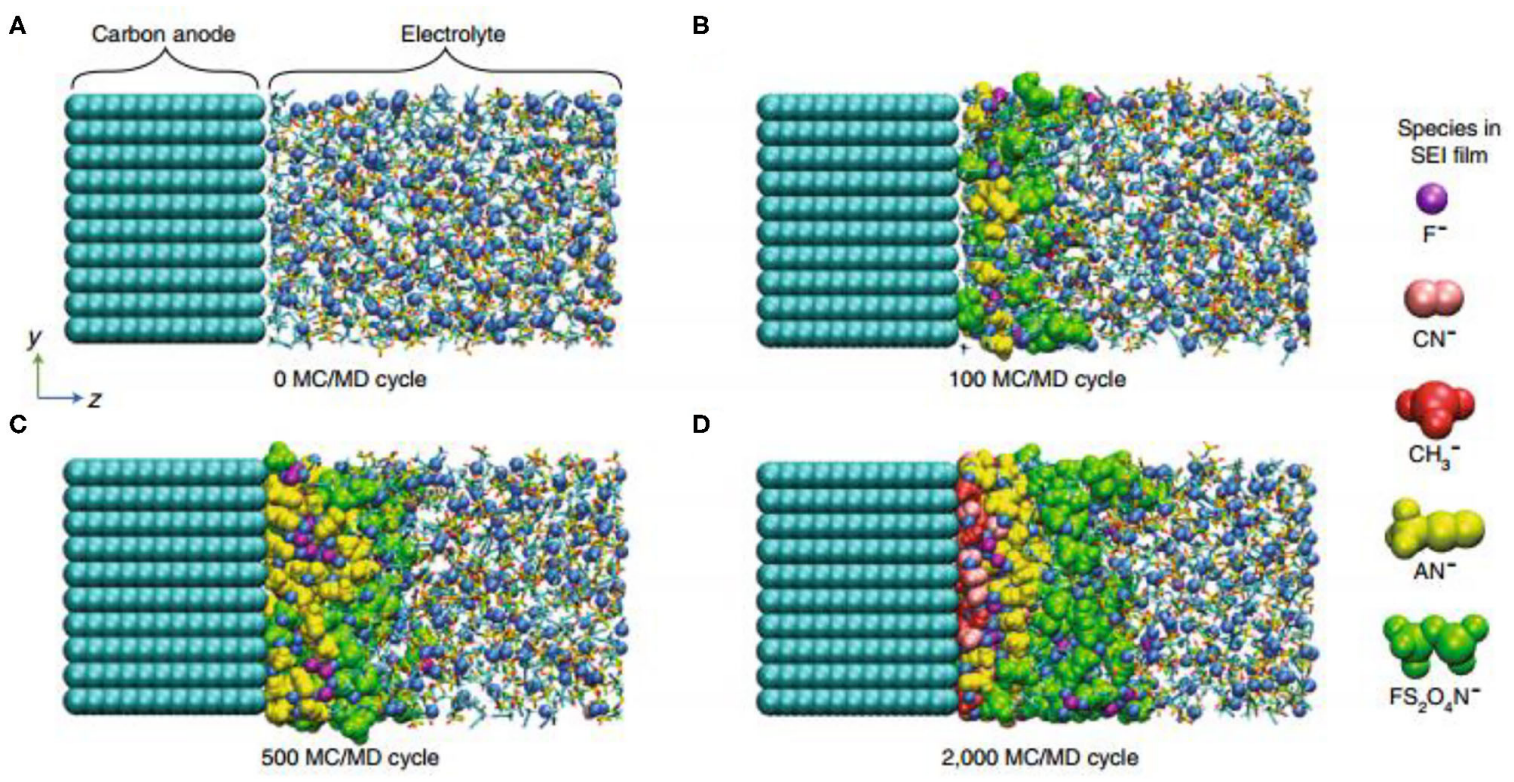
Molecular dynamics simulation of interfacial film formation in high-concentration electrolytes (5 M LiFSA/AN) by the mixed Monte Carlo/molecular dynamics (MC/MD) method: **(A)** Activation process (0 MC/MD cycle). **(B)** Preferential reduction of FSA^−^ to from green FS_2_O_4_N^−^ anions (100 MC/MD cycle). **(C)** Sequential reduction of AN at the FSA^−^-poor surface (500 MC/MD cycle). **(D)** Final equilibrium state to form a stable SEI (2,000 MC/MD cycle) (Takenaka et al., 2018) (reproduced with permission from ACS The Journal of Physical Chemistry C, copyright 2018).

We now present future research directions in this area and address their significance as follows:

1. The formation mechanism of the SEI is in need of in-depth exploration. The majority of literature attributed the preferential reduction of the anion to the decrease in LUMO energy level due to the complexation of the anion with Li^+^ (Figure 6). This proposal represents an important aspect in linking micro-coordination states with electrochemical reactions. Moreover, the equilibrium potential and the solubility of the SEI substance cannot be ignored (Sodeyama et al., 2014; Yamada et al., 2014). The equilibrium reaction potential of the negative electrode (and the positive electrode) is also shifted upwards according to the Nernst equation, because of the increase in lithium-ion activity in the high-concentration electrolyte, which may change the path of the main electrochemical reaction. Also, it is not easy to dissolve the components of the SEI film, because there are fewer free solvent molecules in the high-concentration electrolytic solution (Sodeyama et al., 2014). To tackle the microscopic aspects of the latter, a hybrid Monte Carlo-molecular dynamics approach may be a powerful tool, as recently described in the SEI film formation process. In addition, the modification of electrolyte additives is also an aspect worth studying (Takenaka et al., 2018).
2. Multi-layering is an aspect that should be used to standardize SEI film. Although there are many characterization methods, there is no analysis capable of *in situ* characterization of SEI film (Wang et al., 2016; Yamada et al., 2016).
3. The most challenging task would be to correlate the chemical composition of SEI membranes with their functions (Qian et al., 2015; Wang et al., 2018a).

## Research Methods and Advanced Characterization for CEI Film

The characterization of the interfacial film is very challenging due to many reasons (Chu et al., 2019): First, the reaction of SEI formation is accompanied by lithium-ion insertion and is affected by many factors, including the composition of the electrolyte and the charge and discharge conditions. This means that the chemical composition and structure of the SEI film are complicated. In addition, the thinner SEI is buried between the two electrodes of the battery, and becomes unstable and “non-living” when taken out of the volatile electrolyte in a lithium-ion battery. Since the physical properties of the interfacial film cannot be observed *in situ*, characterizing and understanding the correlation between the interfacial film and battery performance has become extremely important, but remains a challenge to tackle. Moreover, the lack of basic research means that a large number of efforts follow a process of continuous trial and error to study the regulatory role of the interfacial film. In order to optimize the battery's performance, the ideal interfacial film should be thin (to reduce the consumption of active lithium-ions and lower the impedance of the lithium-ion transport process), dense (to better block the electrons), and have a uniform morphology and structure (for uniform lithium-ion transport) and mechanical flexibility (to cushion the volume expansion of the active material).

In addition to some of the traditional characterization methods, recent years have brought along advances and new characterization methods that provided great help for the interpretation of the interfacial film mechanisms with high spatial resolution close to the chemical structural units, and a state close to “effective” interfacial film (Liu et al., 2014; Zhang et al., 2014; Huang et al., 2018). For example, the mechanical curve of the atomic force microscopy is used to test the mechanical elasticity and structure of the interfacial film with nanometer-level resolution, and the non-porous near-field scanning optical microscopy (aNSOM) also uses the AFM tip that is in contact with the surface of the probed material (Ayache et al., 2015a,b). Optical probes for high-resolution spectroscopy and imaging measurements have been used to map the chemical composition of the interfacial film. In addition, secondary time-of-flight ion mass spectrometry (ToF-SIMS) is also used as a detection technique (Zhu et al., 2015; Yuan et al., 2017). Moreover, cryo-STEM, which was originally designed to protect aqueous biological samples from damage, has been introduced since 2017 to study the chemical composition and structure of the interfacial film, and allowed researchers to obtain many exciting results. We will present in the following section some of the novel testing technologies used in the recent years, and mainly discuss their development and some of the progress made in their application for the characterization of surface CEI films (Alvarado et al., 2018; Li et al., 2018a; Zachman et al., 2018).

### Depth Profiling Using *in-situ* TOF-SIMS

**Figure 7:**
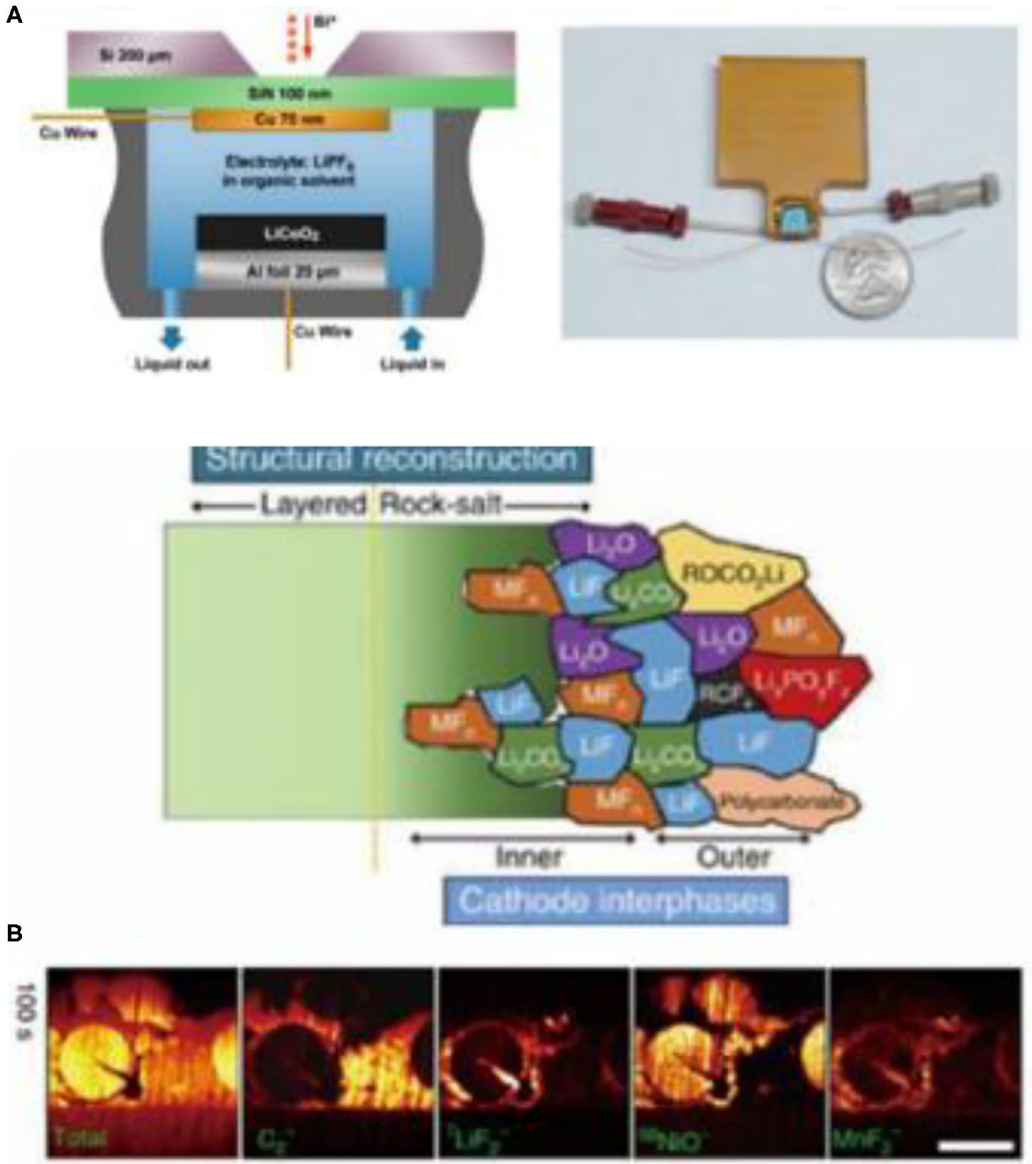
**(A)** Schematic diagram of a device embedded with positive and negative electrodes in liquid battery (left) and picture of the actual device (right) (Zhu et al., 2015) **(B)** Schematic diagram of the composition of the CEI film on the positive electrode surface and the TOF-SIMS ion sputtering distribution at the material interface (Yuan et al., 2017) (reproduced with permission from ACS Nano Letters, copyright 2015).

The application of SIMS for situ in measurements in lithium-ion battery research is still a challenge, due to the high volatility of non-aqueous liquid electrolytes. In order to solve this problem, Zhu et al. designed an *in situ* battery to explore the lithium insertion and extraction process on the copper foil in LiPF_6_/(EC + DMC) electrolyte (Figure 7A; Zhu et al., 2015). In a different study, Li et al. used TOF-SIMS to study the role of the conductive additive in the formation of CEI films and the performance of full cells with nickel-rich layered oxide positive electrodes (LiNi_0.7_Co_0.15_Mn_0.15_O_2_) (Yuan et al., 2017). Area-sensitive SIMS indicated that CEI was originally formed on the conductive carbon additives without electrochemical bias, and that it could passivate the positive electrode particles through the exchange of surface substances (Figure 7B).

Recently, Xu et al. propose new research using molecular eye technology to draw SEI chemical and structural dynamic map. With molecular eye technology, scientists can more accurately understand the inner workings of batteries and find out why they tend to catch fire. According to foreign media reports, researchers from the US Army Combat Capabilities Development Command's Army Research Laboratory and the US Department of Energy's Pacific Northwest National Laboratory explore how a chemical reaction occurs when two key parts of a battery come into contact, and form a key part of the battery, commonly known as a SEI film (Zhou et al., 2020).

Scientists at the Environmental Molecular Sciences Laboratory and the Pacific Northwest National Laboratory have developed *in-situ* liquid secondary ion mass spectrometry. In collaboration with Army scientists, they used this technology to study how electrode-electrolyte interface chemistry works at the molecular level when the battery is charged for the first hour. By monitoring the formation of SEI and its chemical changes, a picture of the chemical reactions that occurred are drawm, and combining molecular dynamics simulation methods, their work revealed something that could only have been speculated before (Zhou et al., 2020).

### Detection of Spatially Resolved SEI Chemical Structures Using Nano-Infrared Spectroscopy

Usually, FTIR and Raman vibrational spectroscopy are used to analyze the composition and spatial distribution of the SEI film, but their spatial resolution is usually limited by the beam size, which is mainly in the order of decimeters. However, it is well-known that the composition of the interfacial film at the nanoscale is highly uneven. Therefore, chemical analysis results obtained for SEI film by FTIR are only based on the average value of the light emitting area. In order to solve this problem, researchers in recent years have invented a method of infrared spectrum imaging at nanometer-scale spatial resolution. Through three different methods, including infrared non-aperture near field scanning optical microscopy (IR-aNSOM) (Wickramasinghe and Williams, 1994; Centrone, 2015; Muller et al., 2015), light-induced resonance (PTIR) (Dazzi and Prater, 2017), and light-induced force microscopy (PiFM) (Liu et al., 2018). Because of the unique capabilities of these high-resolution chemical imaging methods, new insight can be gained in the study of interfacial films.

In aNSOM, a beam of light is fixed on a metal AFM probe placed close to the sample surface to allow the light intensity to be greatly enhanced at the probe tip due to local surface plasmon resonance. The scattered signal is modulated by the local dielectric properties of the sample to be measured and collected from the far field, where different compounds absorb the infrared spectrum, thereby producing image contrast in the IR-aNSOM as the AFM probe scans the entire surface. Based on this, Ayache et al. (2015b) pioneered the application of IR-aNSOM in SEI research on Sn and HOPG electrodes. In their first study, the HOPG electrode was selected from OCP (~2.9 V) to 0.8 V (in Li ion embedded graphite) in 1.0M LiPF_6_ dissolved in EC / DEC (weight ratio of 1:2) Samples taken from the electrolyte at 1.66, 1.36, and 0.9V were used for IR-aNSOM measurement of the (002) surface. As a result, these researchers reported that the morphological image of the sample discharged to 1.6 V showed that the 200 nm SEI layer was composed of loosely packed particles, while the multi-wavelength near-field IR absorption image showed a large contrast, indicating that SEI has a relatively high The thick inner and outer particle layers, as well as the two layers, have unique but uniform composition.

These characterization methods are widely used for characterizing SEI films on the surface of negative electrode. Considering that the positive electrode interface film is thin, it may not be particularly widely used. In the future, research on the positive electrode interfacial films can also be used to characterize the composition and dynamic characteristics of interfacial films by analogy.

### Original Structure of Interfacial Films Characterized by Cryo-TEM

**Figure 8:**
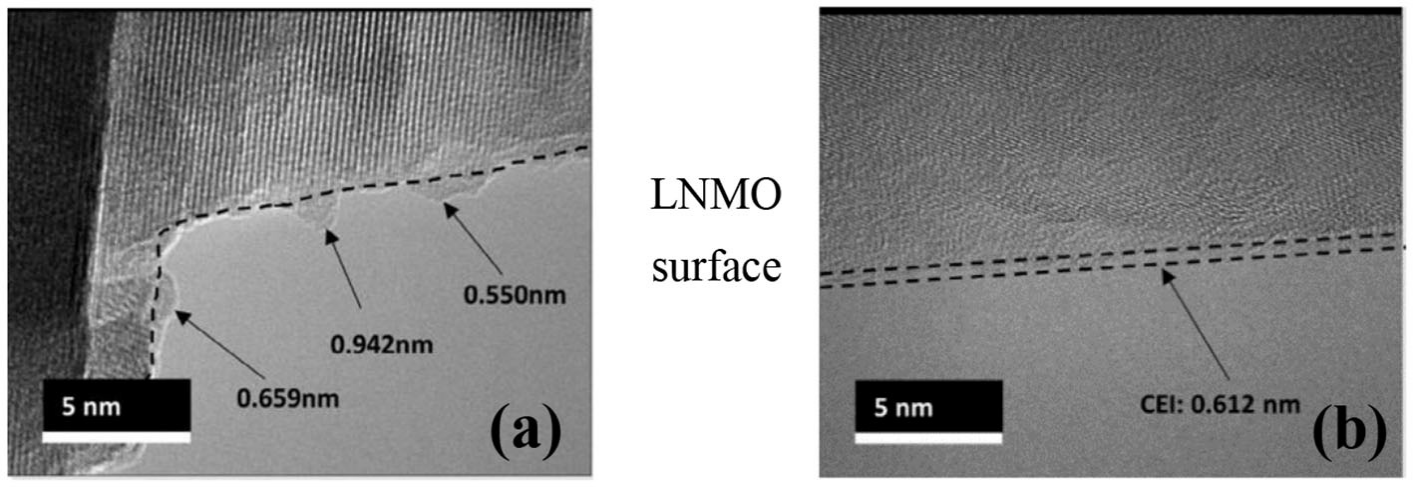
**(a)** Cryo-TEM images of LNMO particle CEIs after discharging for 50 cycles with 1.2 M LiPF_6_/(EC + EMC, 3:7) and **(b)** 3 M LiFSI-SL (Alvarado et al., 2018).

Although traditional transmission electron microscopy (TEM) can obtain atomic-level structural images of the material, it is still not ideal for the characterization of interfacial films, because these films are chemically active and sensitive to electron beam radiation. Therefore, many studies on interfacial films obtained from traditional TEM are now limited to the decimeter level because detailed crystal structure cannot be probed. However, recent studies have used cryo-electron microscopy to study the interfacial film at the atomic level, maintaining the original structure by freezing samples in liquid nitrogen (Li et al., 2017, 2018a,b; Alvarado et al., 2018; Wang et al., 2018b). In the field of characterization of high-voltage electrolytes for lithium-ion batteries, Alvarado et al. used cryo-electron microscopy (cryo-STEM) to retain the structure of the CEI film of the LiNi_0.5_Mn_1.5_O_4_ cathode and avoid disturbance to the electron transfer (Alvarado et al., 2018). The technique probes obvious changes in thickness and uniformity, while the CEI film of the electrolyte system under study remains thin and uniform (Figure 8). Recently, Huang used Cryo-STEM to study the spatial distribution of SEI components such as LiF and Li_2_O in the SEI of the Li metal anode. Cryo-STEM EELS confirmed that the dense SEI connected with the negative electrode material does not contain LiF (William et al., 2020).

### Analysis of Mechanical Elasticity of SEI Films by AFM Mechanical Curves

In addition to being an electronic insulator and ion conductor, the SEI/CEI layer also needs to have sufficient mechanical flexibility and excellent hardness to adapt to the changes in the electrode volume during charge and discharge cycles, especially for conversion reactions with large volume expansion, as with alloy anodes and some cathode materials. A fragile SEI/CEI film will break during the volume expansion of the electrode and cause the formation of a new interface film, which will result in lower coulombic efficiency and rapid capacity decay. Although many instruments are used to detect the chemical composition and morphology of the interfacial film, the characterization of its mechanical properties is still relatively unaddressed. To solve this problem, AFM-based mechanical instruments, local strain forces, or “nano surveys” measurements were proposed as the only suitable equipment (Chu et al., 2019). Liao et al. (2018) used AFM to characterize the roughness of the CEI film formed by the LiDFBOP additive in graphite-LiNi_0.5_Co_0.2_Mn_0.3_O_2_ system. The results showed that the CEI film formed by LiDFBOP on the positive electrode LiNi_0.5_Co_0.2_Mn_0.3_O_2_ was more uniform than that in transition electrolyte system without additive. This also provides a basis for LiDFBOP to adjust the positive electrode interface mechanism, and thereby improve the electrochemical performance of the system.

## Outlook

In this article, we reviewed the studies that addressed the composition and properties of the interfacial film on the positive electrode of lithium-ion batteries over the past decade. It can be seen from various studies that researchers have been paying increasing attention to the investigation of the CEI membranes, and obtaining informative results that will guide the research on the anode decay mechanism and the design and optimization of electrolyte formulation. The interfacial film can be seen as a phenomenon associated with the reaction between the electrolyte and the electrode material, it can also be used as a bridge toward studying the reaction mechanism. It is necessary to balance the relation between the loss of irreversible capacity and the repair during the formation of the CEI film, and whether the consumed lithium-ions can be added to the deteriorated performance with time.

The use of advanced characterization methods enables us to explore the key to the film formation mechanism. The application of some of the most advanced characterization methods reviewed in this article is not very extensive, and these characterization methods suffer from few problems that require consideration by future researchers. First, there is a need for the development of proper *in situ* characterization protocols for the battery. Second, multiple detection technologies may need to be combined to achieve the characterization of multiple properties of the interface. Finally, proper methodologies are needed to perform the characterization of the positive electrode interfacial film while the electrode is paired with the negative electrode in a full cell, to study the film formation kinetics of the entire process.

Our current research on the interface films is largely traceable to the study of composite electrodes containing conductive agents and binders. This will affect the formation of interfacial films, and lead to misunderstandings of its composition. On the other hand, the simultaneous observation of the same decomposition products on the negative and positive electrodes is more confusing, because different reactions will be involved on each side of the electrode. In order to avoid these complications, it is necessary to study the surface mode of the electrode, such as oxidized microspheres and films, which will help study the activity between the electrolyte and the surface of the individual material. Another obstacle is the lack of information on the kinetic properties of the surface film formed at the interface. The implementation of *in-situ* technologies such as *in-situ* XPS, XAS, and surface-enhanced Raman spectroscopy, may draw a clear portrait of the surface structural features that are well-defined in the working environment. An example is seen in the recent use of high-voltage X-ray photoelectron spectroscopy to study the latest advances in interface films on silicon electrodes.

Overall, researchers around the world have invested considerable effort into the research of interfacial film, and more work is expected in this field in the future. The secret of the relation between the interfacial film and the device electrochemical performance is being gradually unveiled with the expansion of research and the advancement of characterization methods.

## Author Contributions

DZ: conceptualization, methodology, and writing–original draft. SL: writing–review & editing, funding acquisition, and project administration. All authors contributed to the article and approved the submitted version.

## Conflict of Interest

The authors declare that the research was conducted in the absence of any commercial or financial relationships that could be construed as a potential conflict of interest.
